# Efficacy of a Decision Aid in Breast Cancer Patients Considering Immediate Reconstruction: Results of a Randomized Controlled Trial

**DOI:** 10.1097/PRS.0000000000011100

**Published:** 2023-10-09

**Authors:** Jacqueline A. ter Stege, Leonie A. E. Woerdeman, Jacobien M. Kieffer, Kerry A. Sherman, Joost A. Agelink van Rentergem, Frederieke H. van Duijnhoven, Martine A. van Huizum, Miranda A. Gerritsma, Marianne Kuenen, Eveline M. L. Corten, Nikola (A. N.) Kimmings, Quinten (P. Q.) Ruhé, Irene S. Krabbe-Timmerman, Martijne van’t Riet, Daniela E. E. Hahn, Arjen J. Witkamp, Hester S. A. Oldenburg, Eveline M. A. Bleiker

**Affiliations:** Amsterdam, Rotterdam, Amersfoort, Leeuwarden, Delft, Utrecht, and Leiden, the Netherlands; and Sydney, New South Wales, Australia; From the 1 Division of Psychosocial Research and Epidemiology; 2 Department of Plastic and Reconstructive Surgery; 4 Department of Surgical Oncology; 11 Department of Psychosocial Counseling; 14 Family Cancer Clinic, Netherlands Cancer Institute, Antoni van Leeuwenhoek; 3 Centre for Emotional Health, School of Psychological Sciences, Macquarie University Sydney; 5 Plastic and Reconstructive Surgery, Erasmus Medical Center; 6 Plastic and Reconstructive Surgery, Franciscus Gasthuis & Vlietland; 7 Surgery, Slotervaart Medical Center; 8 Plastic and Reconstructive Surgery, Meander Medical Center; 9 Plastic and Reconstructive Surgery, Medical Center Leeuwarden; 10 Department of Surgery, Reinier de Graaf Gasthuis; 12 Department of Surgery, University Medical Center Utrecht; 13 Department of Clinical Genetics, Leiden University Medical Center.

## Abstract

**Background:**

Breast cancer patients face complex decisions about immediate breast reconstruction (BR) after mastectomy. The authors evaluated the efficacy of an online decision aid in improving the decision-making process, decision quality, and health outcomes in breast cancer patients considering immediate BR.

**Methods:**

In a multicenter, randomized, controlled trial, patients were allocated to either the intervention group, receiving care as usual with access to an online decision aid, or the control group, receiving care as usual with an information leaflet. The primary outcome was decisional conflict. Secondary outcomes assessed the process of decision-making (eg, preparation for decision-making, satisfaction with information), decision quality (decision regret, knowledge), and health outcomes (eg, satisfaction with BR outcomes, body image). Patients completed questionnaires at time (T) 0 (baseline); T1 (1 week after consultation with a plastic surgeon); and T2 (3 months) and T3 (12 months) after surgery.

**Results:**

The authors included 250 patients. Decisional conflict decreased over time in both groups, with no between-group differences. Intervention participants felt better prepared for decision-making than controls (*P* = 0.002). At T2, 87% of intervention participants were very satisfied with the information about BR, compared with 73% of control participants (*P* = 0.011). No significant between-group differences were observed in any other outcome.

**Conclusions:**

The authors’ online decision aid was as effective in reducing decisional conflict as an information leaflet about immediate BR after mastectomy. However, the decision aid substantially improved the decision-making process by better preparing breast cancer patients for decisions about immediate BR.

**CLINICAL QUESTION/LEVEL OF EVIDENCE:**

Therapeutic, II.

In Western European countries, approximately 1 in 7 women develops breast cancer.^1^ As surgical treatment, approximately 60% to 70% of all breast cancer patients undergo breast-conserving surgery,^2–4^ whereas 30% to 40% undergo mastectomy.^2–5^ Mastectomy can especially have a negative impact on psychosocial outcomes such as body image and sexual functioning.^6–9^ To restore breast contour, and potentially improve psychosocial outcomes after mastectomy, women may opt for breast reconstruction (BR). Breast reconstructive surgery can be performed immediately after mastectomy (IBR), or BR can be delayed. In addition, there are several modes of BR (implant-based, autologous, and a combination of both). All BR options have their pros and cons. Personal values and preferences of patients play an important role in the decisions about BR.^10,11^

Dutch guidelines recommend discussing the possibility of IBR with every patient before mastectomy.^12^ The number of women choosing BR, and especially IBR, is increasing.^2,13–18^ In 2021, 29% of patients undergoing a mastectomy opted for IBR in the Netherlands.^19^ Approximately 10% of patients opted for delayed BR.^20–22^ However, both nationally and internationally, immediate BR rates vary substantially across hospitals and geographic locations, ranging from 0% to 77% among Dutch hospitals.^18,23–25^

Decision-making regarding BR is complex, and can be challenging for women, especially so soon after receiving a breast cancer diagnosis.^11^ Previous studies highlight the importance of providing qualitative and realistic preoperative information and decisional support to enable women to make a long-term satisfying decision about BR.^26–33^ Although most women are satisfied with their reconstructed breast, and decision regret is generally low,^34^ a minority of women experience mild to moderate regret.^26,35^ Poor knowledge of BR coupled with feelings of being poorly prepared to make a decision are commonly experienced and are linked to poor outcomes, like decision regret.^26,36–38^

Patient decision aids (pDAs) are tools developed to support the process of shared decision-making between patients and physicians.^39^ They explicitly describe the decision that patients face, provide evidence-based information about treatment options including their pros and cons, and provide support in clarifying personal values relevant to the decision.^39^ Patient decision aids for a variety of treatment decisions have been shown to reduce decisional conflict and increase knowledge and insight into personal values related to the decision.^40,41^

Worldwide, few interventions to support patient decision-making about BR are available.^42^ A systematic review assessing the effectiveness of these interventions found that patient satisfaction and involvement in decision-making improved following pDA exposure; nevertheless, results on other outcomes were mixed. However, most studies were methodologically flawed (eg, small sample size, single-center design), and neglected to control for potential confounding variables such as complications.^42,43^

To support women in making an informed decision regarding IBR following mastectomy, and in the absence of any decision-making supportive interventions for the Dutch population, we developed an online pDA. The primary aim of this study was to evaluate the efficacy of this pDA in reducing decisional conflict and address limitations of prior studies by including a large sample size and using a multicenter randomized controlled design.^42,43^ As a secondary aim, we evaluated the impact of the pDA on the decision-making process, decision quality, and patient-reported health outcomes.

## PATIENTS AND METHODS

### Design

We conducted a 2-arm randomized controlled trial in 8 hospitals throughout the Netherlands. A detailed description of the study protocol is published elsewhere,^44^ and the trial protocol was registered. Group allocation was by means of simple randomization (1:1) and stratified by site and by patients’ surgical treatment options (ie, [1] the patient opted for mastectomy while eligible for both mastectomy and breast conserving surgery, or [2] the patient opted for mastectomy and was eligible for mastectomy only). The institutional review boards of all participating hospitals approved the study.

### Eligibility Criteria

Patients were eligible if they were (1) a female patient at least 18 years old, (2) diagnosed with breast cancer or ductal carcinoma in situ, (3) scheduled to undergo mastectomy and eligible for IBR, and (4) had been referred to a plastic surgeon. The consultation with the plastic surgeon was scheduled at least 3 days after study invitation to allow sufficient time for participants to complete informed consent, the baseline questionnaire, and the pDA or the information leaflet before their consultation. In addition, patients were required to have (5) internet access and basic computer skills, and (6) sufficient command of the Dutch language.

### Procedure

Patients were invited for study participation by their treating surgeon or nurse during the consultation in which the possibility of BR was discussed. After completing the informed consent form and baseline questionnaire, participants were allocated randomly to the intervention or the control group. Intervention group participants received a link to the pDA and control group participants received an information leaflet on BR by e-mail. Participants completed questionnaires at time (T) 0 (baseline), T1 (1 week after consultation with the plastic surgeon), T2 (3 months after surgery), and T3 (12 months after surgery). Intervention group participants had unlimited access to the pDA during the study (see the study protocol for full details).^44^

### Intervention Group

Patients in the intervention group received care as usual (CAU) and access to the online interactive pDA (named “breast reconstruction patient decision aid,” available at https://br.keuzehulp.nl [in Dutch]). The pDA aims to prepare patients for consultation with a plastic surgeon. It contains evidence-based information about BR options, the pros and cons of each option, value clarification exercises, and patient stories of women who previously faced the decision. It results in a summary sheet including a patient’s BR preferences to discuss with their plastic surgeon. The information is tailored to the patient’s treatment options relevant for decision-making about BR (see the development paper^45^ for full details of the pDA).

### Control Group

Patients in the control group received CAU and an information leaflet about BR, typically provided as standard in Dutch hospitals.^46^ The 39-page leaflet provides information about all types of BR, including drawings and photographs of results. In contrast to the pDA, the leaflet is not structured to guide decision-making; is not tailored to the patient’s treatment options; and does not contain value clarification exercises, patient stories, or a summary sheet including a patient’s BR preferences.

### Study Measures

At baseline, sociodemographic and clinical information was obtained in addition to patients’ preference regarding BR, preferred involvement in decision-making about BR,^47^ frequency of and skills regarding internet use, and information coping style.^48^ Information about patients’ surgical treatment, complications, and adjuvant treatment was obtained by means of postsurgical questionnaires (at T2 and T3). Standardized self-report questionnaires were administered to assess the primary and secondary outcomes (Table 1).^1–75^ The primary outcome was decisional conflict measured by the Decisional Conflict Scale,^49–51^ assessing how well informed patients feel about their decision, the level of uncertainty about the best choice, and the perceived effectiveness of decision-making. Secondary outcomes included the decision-making process measured by satisfaction with information,^52^ satisfaction with the plastic surgeon,^52^ preparedness for decision-making,^53,54^ patients’ perceived levels of shared decision-making during consultation with their plastic surgeon,^55,56^ and patients’ perceived level of involvement in decision-making.^47^ Decision quality was measured by knowledge of BR^44^ and by decision regret.^57,58^ Patient-reported health outcomes included patients’ actual choice regarding BR, patient satisfaction with breast,^52^ satisfaction with reconstruction outcomes,^52^ body image,^59^ sexual functioning,^59^ breast symptoms,^59^ and anxiety.^60^

**Table 1.** Overview of Primary and Secondary Outcome Measures

Outcome Measure	Instrument	Details	T0	T1	T2	T3
Primary outcome						
Decisional conflict	DCS^49,51^	The DCS has five subscales (uncertainty, feeling informed, feeling clear about values, feeling supported, and effective decision-making) and a total score. Score range: 0–100, higher scores indicate more decisional conflict. Scores >37.5 are associated with decision delay and feeling unsure about implementation.^49,51^ The effective decision-making subscale was not assessed at T0, as items of this scale were considered inappropriate to assess before patients had a consultation with a plastic surgeon. As an alternative for the total score, the combined score without effective decision-making subscale was calculated by summing items of the other 4 subscales, dividing by 12, and multiplying by 25.^72,73^	X	X	X	X
Secondary outcome						
Decision-making process						
Satisfaction with information	2 study-specific questions Satisfaction with information subscale of BREAST-Q^52^	How satisfied are you with the information about BR? How satisfied are you with the information in the decision aid/information leaflet? Score range: 0–100, higher scores indicate higher satisfaction. Subscale is assessed only in women who had BR.			X	X
Satisfaction with plastic surgeon	Satisfaction with the plastic surgeon subscale of BREAST-Q^52^	Score range: 0–100, higher scores indicate higher satisfaction.		X		
Preparedness for decision-making	Preparation for decision-making ccale^53,54^	Score range: 0–100, higher scores indicate higher perceived level of preparation for decision-making.		X		
Shared decision-making	Shared Decision-Making Questionnaire (SDM-Q-9)^55,56^	Score range: 0–100, higher scores indicate higher levels of perceived shared decision-making.		X		
Patient involvement in decision-making	Control Preferences scale^47^	1 item, 5-point Likert-type scale categorized as active (A, B), collaborative (C), or passive (D, E), with the following answer categories: (A) I made the decision about BR alone, (B) I made the decision about BR after seriously considering my physician’s opinion, (C) my physician and I made the decision about BR together, (D) my physician made the decision about BR after seriously considering my opinion, (E) my physician made the decision about BR alone.		X		
Decision quality						
Knowledge of BR	Study-specific questionnaire, translated, and adapted from a questionnaire used in prior research^74^	10 items answered with true/false/I don’t know. The total score is the number of correctly answered items, score range: 0–10. Items cover contraindications, risk factors, duration of the recovery period, impact on sensation, number of surgical procedures required, complexity of flap- vs. implant-based BR, risk for complications, impact on breast cancer treatment and survival rates, and the opportunity to spare the nipple.	X	X	X	X
Decision regret	Decision Regret Scale (DRS)^57,58^	Score range: 0–100, higher scores indicate greater regret. A score ≥ 30 means that a participant responded that she was more or less in agreement with at least one of the statements about an experience of regret.^75^			X	X
Patient-reported health outcomes						
Actual choice	Study-specific questions	The choice whether or not a patient had immediate BR, and the type of BR (tissue-expander, implant, autologous tissue, or a combination of an implant and autologous tissue).			X	X
Satisfaction with breasts	Satisfaction with breasts subscale of the BREAST-Q^52^	This scale measures body image in terms of a woman’s satisfaction with her breast. Items cover breast appearance, and satisfaction with breasts in relation to how a bra fits and how the breasts look when clothed or unclothed. Score range: 0–100, higher scores indicate higher satisfaction.			X	X
Satisfaction with reconstruction outcome	Satisfaction with breast outcome subscale of the BREAST-Q^52^	This scale measures a woman’s overall appraisal of the outcome of her breast surgery. Items cover whether woman’s expectations were met with respect to the aesthetic outcome and the impact surgery has had on her life and the satisfaction with the decision to have breast reconstructive surgery. Score range: 0–100, higher scores indicate higher satisfaction. Subscale is assessed only in women with BR only.			X	X
Body image	Body image subscale of the EORTC QLQ-BR23^59^	Score range: 0–100, higher scores indicate higher body image.			X	X
Sexual functioning	Sexual functioning subscale of EORTC QLQ-BR23^59^	Score range: 0–100, higher scores indicate higher sexual functioning.			X	X
Sexual enjoyment	Sexual enjoyment item of the EORTC QLQ-BR23^59^	Score range: 0–100, higher scores indicate higher sexual enjoyment.			X	X
Breast symptoms	Breast symptoms subscale of the EORTC QLQ-BR23^59^	Score range: 0–100, higher scores indicate higher levels of breast symptoms.			X	X
Anxiety	State scale of the State-Trait Anxiety Inventory (STAI-6)^60^	Score range: 20–80, higher scores indicate higher levels of anxiety.	X	X	X	X

DCS, Decisional Conflict Scale.

### Statistical Analyses

Data were pseudonymized before analysis. Missing values were either handled according to published scoring algorithms, or replaced by the mean score of completed items within the scale or subscale for each individual, provided that a minimum of 75% of scale or subscale items was completed. Appropriate tests were used to compare continuous and categorical baseline characteristics between groups.

We used a mixed modeling approach to compare outcomes between groups over time. For outcomes measured at all 4 time points, we used random intercept and slope models with linear and quadratic time effects to determine whether an initial change in the outcome was maintained during follow-up (time was included as weeks since baseline). For outcomes without a baseline assessment, we used time to follow-up analyses (ie, the remaining measurement occasions were introduced as a categorical variable). For categorical outcomes, generalized linear models were used.

In all above models, we adjusted for hospital, body mass index (BMI), and potential nonignorable dropout on the basis of the Akaike information criterion and the bayesian information criterion.^61,62^ In the analyses of outcomes only assessed in participants who had BR (ie, BREAST-Q subscales satisfaction with information and satisfaction with reconstruction outcome), we included history of breast cancer and baseline anxiety in the model selection procedure because of significant baseline differences between the intervention and control groups in this subset.

The differences in mean change scores over time and in mean scores between groups were accompanied by standardized effect sizes (ESs). ESs of 0.20 were considered small; 0.50, moderate; and 0.80, large.^63^ An ES greater than or equal to 0.50 was considered clinically relevant.^64^ To limit type 1 errors because of multiple testing, a value of *P* = 0. 01 was considered statistically significant. Analyses were performed on an intention-to-treat basis.

## RESULTS

**Fig. 1.**
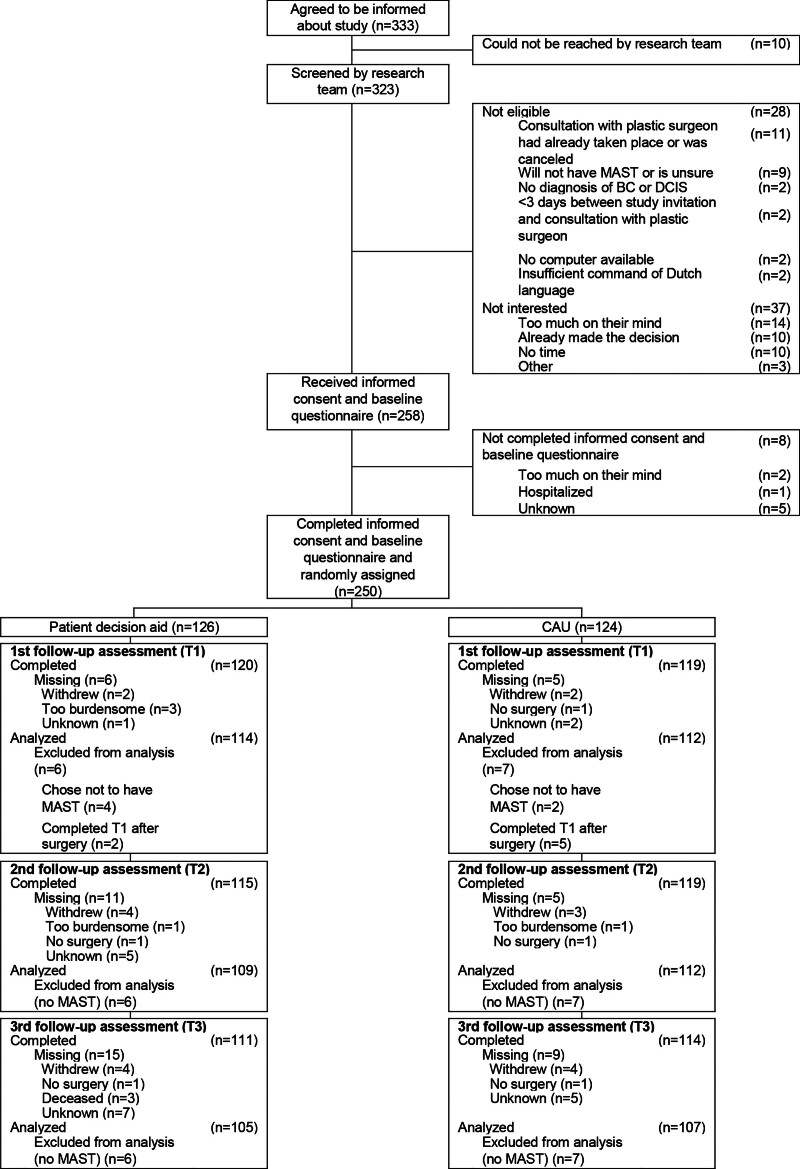
Consolidated Standards of Reporting Trials diagram. *MAST*, mastectomy; *BC*, breast cancer; *DCIS*, ductal carcinoma in situ.

Patients were recruited between August of 2017 and April of 2019, and follow-up was completed in November of 2020. See Figure 1 for participant flow. In total, 333 patients were informed about the study. Of these patients, 250 patients completed informed consent and baseline questionnaire and were assigned randomly to either the intervention (*n* = 126) or the control (*n* = 124) group. Follow-up assessments were completed by 96%, 94%, and 90% of the participants, at T1, T2, and T3, respectively. Completion and inclusion rates of follow-up assessments did not differ significantly between groups.

Participants had an average age of 50.1 years. More than half of the participants (51.6%) were highly educated, and most (93.2%) were born in the Netherlands. All baseline sociodemographic and clinical characteristics were balanced between both groups, except for BMI. Intervention participants were more often obese than control participants (BMI ≥30 kg/m^2^; *P* = 0.01) (Table 2).

**Table 2.** Background Characteristics of Participants

Characteristic	All Patients	Intervention Group (%)	Control Group (%)	*P*
No.		126	124	
Age, yr				0.64
Mean	50.1	50.4	49.8	
SD	11.0	11.0	11.1	
Educational level^a^				0.81
Low	10 (4.0)	5 (4.0)	5 (4.0)	
Intermediate	109 (43.6)	57 (45.2)	52 (41.9)	
High	129 (51.6)	62 (49.2)	67 (54.0)	
Missing	2 (0.8)	2 (1.6)	0 (0.0)	
Born in the Netherlands	233 (93.2)	118 (93.7)	115 (92.7)	0.78
Married or in a relationship	214 (85.6)	111 (88.1)	103 (83.1)	0.72
Children (yes)	199 (79.6)	101 (80.2)	98 (79.0)	0.83
Body mass index				0.01^d^
<30 kg/m^2^	219 (87.6)	104 (82.5)	115 (92.7)	
≥30 kg/m^2^	31 (12.4)	22 (17.5)	9 (7.3)	
Smoker (yes)	14 (5.6)	8 (6.3)	6 (4.8)	0.60
Comorbidities				0.56
0	128 (51.2)	65 (51.6)	63 (50.8)	
1	79 (31.6)	37 (29.4)	42 (33.9)	
≥2	42 (16.8)	24 (19.0)	18 (14.5)	
Missing	1 (0.4)	0 (0.0)	1 (0.8)	
Diagnosis				0.18
Invasive BC	151 (60.4)	69 (54.8)	82 (66.1)	
DCIS	62 (24.8)	35 (27.8)	27 (21.8)	
Both	37 (14.8)	22 (17.5)	15 (12.1)	
Bilateral diagnosis	12 (4.8)	5 (4.0)	7 (5.6)	0.54
Time since diagnosis, wk^b^				0.73
Median	3	3	4	
IQR	18	17	18	
Diagnosis in irradiated breast(s)	27 (10.8)	10 (7.9)	17 (13.7)	0.14
Genetic predisposition or familial increased risk for BC				0.86
No	153 (61.2)	75 (59.5)	78 (62.9)	
Yes	40 (16.0)	21 (16.7)	19 (15.3)	
I don’t know	57 (22.8)	30 (23.8)	27 (21.8)	
Neoadjuvant therapy	91 (36.4)	41 (32.5)	50 (40.3)	0.20
Chemotherapy	86 (34.4)	39 (31.0)	47 (37.9)	
Endocrine therapy	9 (3.6)	5 (4.0)	4 (3.2)	
Immunotherapy	23 (9.2)	10 (7.9)	13 (10.5)	
Indication for adjuvant radiotherapy				0.39
No	71 (28.4)	30 (23.8)	41 (33.1)	
Yes	61 (24.4)	31 (24.6)	30 (24.2)	
Maybe	75 (30.0)	42 (33.3)	33 (26.6)	
I don’t know	43 (17.2)	23 (18.3)	20 (16.1)	
Diagnosis of BC/DCIS in the past				0.46
No	210 (84.0)	108 (85.7)	102 (82.3)	
Yes	40 (16.0)	18 (14.3)	22 (17.7)	
Prior breast surgery for BC/DCIS				
Breast conserving surgery	32 (12.8)	15 (11.9)	17 (13.7)	0.67
Mastectomy^c^	9 (3.6)	4 (3.2)	5 (4.0)	0.72
Mastectomy without BR	4 (1.6)	0 (0.0)	4 (3.2)	
Mastectomy with BR	5 (2.0)	4 (3.2)	1 (0.8)	
BR preference^c^				0.23
Strong for BR	143 (57.2)	75 (59.5)	68 (54.8)	
Slight for BR	51 (20.4)	21 (16.7)	30 (24.2)	
No preference	33 (13.2)	21 (16.7)	12 (9.7)	
Slight for no BR	9 (3.6)	4 (3.2)	5 (4.0)	
Strong for no BR	14 (5.6)	5 (4.0)	9 (7.3)	
Patients’ preferred involvement in decision-making about BR				0.25
Active	127 (50.8)	69 (54.8)	58 (46.8)	
Collaborative	104 (41.6)	46 (36.5)	58 (46.8)	
Passive	19 (7.6)	11 (8.7)	8 (6.5)	
How often do you use the internet?^c^				0.60
(Almost) daily	224 (89.6)	114 (90.5)	110 (88.7)	
About once or several times per week	24 (9.6)	12 (9.5)	12 (9.7)	
Less than once per week	2 (0.8)	0 (0.0)	2 (1.6)	
How well can you use the internet?^c^				0.39
Very well	184 (73.6)	90 (71.4)	94 (75.8)	
Average	65 (26.0)	36 (28.6)	29 (23.4)	
Very bad	1 (0.0)	0 (0)	1 (0.8)	
Monitoring coping style (TMSI)				0.85
Mean	38.2	38.1	38.3	
SD	7.8	7.7	7.9	
Blunting coping style (TMSI)				0.76
Mean	34.0	34.1	33.9	
SD	6.3	6.2	6.4	

BC breast cancer; IQR interquartile range; DCIS ductal carcinoma in situ; BR breast reconstruction; TMSI Threatening Medical Situations Inventory.

a Low = primary school, lower vocational; intermediate = secondary school, intermediate vocational; high = higher vocational, university.

b Based on Mann-Whitney test.

c Based on Fisher exact test.

d Statistically significant.

There were no differences between intervention and control groups in the number of participants with adjuvant treatment, surgical complication(s), and loss of BR as a consequence of complication(s). (**See Table, Supplemental Digital Content 1**, which shows group differences in adjuvant treatment and complications of breast surgery, http://links.lww.com/PRS/G976.)

Among intervention group participants, 95.6% reported that they used the pDA, of whom 52.8% reported that they discussed the pDA summary sheet with their plastic surgeon. Among control group participants, 96.4% reported that they used the information leaflet.

### Primary Outcome

**Fig. 2.**
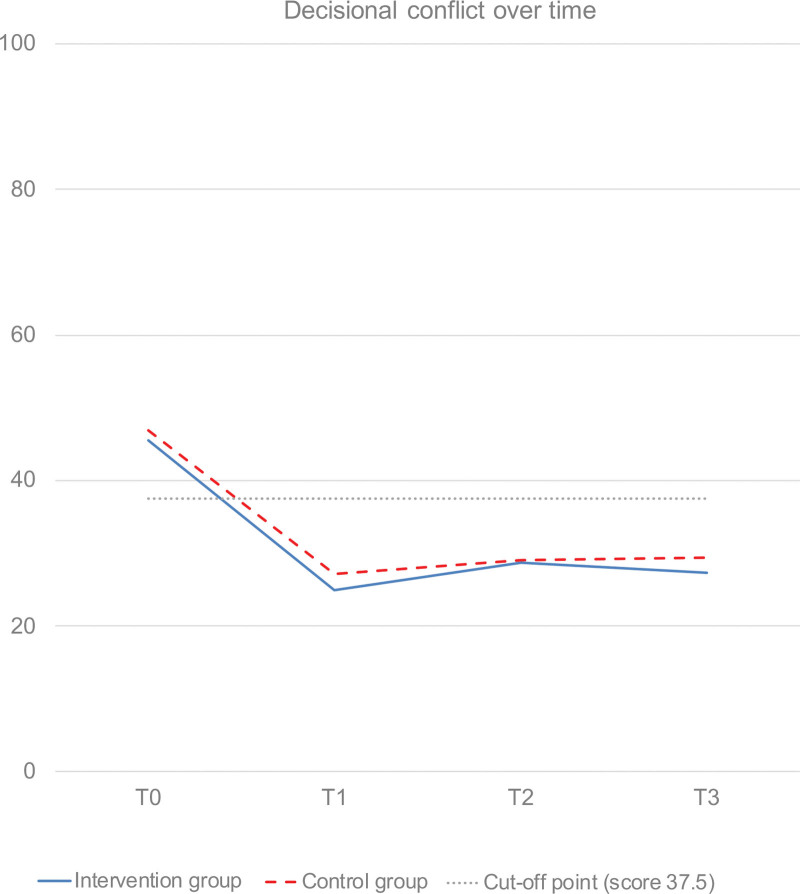
Change over time in decisional conflict (combined score without effective decision-making subscale). Cutoff point at score 37.5: scores exceeding 37.5 are associated with decision delay and feeling unsure about implementing decisions.

There were no significant differences between the intervention group and the control group in decisional conflict over time (Tables 3 and 4 and Fig. 2). In both groups, decisional conflict significantly decreased from baseline to T1, and remained stable thereafter. (**See Table, Supplemental Digital Content 2**, which shows the effects of time on the primary outcome, http://links.lww.com/PRS/G977.) At T1, 13.4% of participants had clinically significant decisional conflict (score >37.5) (no between-group difference: chi-square = 0.80, *P* = 0.371).

**Table 3.** Group Differences in Decisional Conflict (Primary Outcome) over Time^a,b^

	T0	T1^c^	T2^c^	T3^c^	Group × Time Effect		ES^d^		
	Mean (SD)	Mean (SD)	Mean (SD)	Mean (SD)	β (SE)	*P*	T0–T1	T0–T2	T0–T3
Combined score without effective decision-making subscale^e^					−0.00 (0.17)	0.978	−0.06	0.06	−0.05
Intervention group^f^	45.50 (15.25)	25.02 (15.01)	28.26 (15.41)	27.16 (15.37)					
Control group	46.88 (15.23)	27.33 (15.51)	28.63 (18.14)	28.93 (17.81)					
Uncertainty subscale					−0.23 (0.21)	0.264	−0.02	0.14	0.08
Intervention group^f^	47.69 (28.88)	27.80 (21.58)	32.48 (24.17)	31.73 (22.82)					
Control group	49.13 (26.33)	29.46 (21.49)	29.76 (22.59)	30.14 (23.61)					
Feeling informed subscale^g^					0.01 (0.22)	0.966	0.07	0.08	−0.03
Intervention group^f^	48.08 (22.34)	22.57 (18.59)	25.15 (17.69)	24.12 (18.58)					
Control group	50.54 (22.21)	23.44 (16.72)	26.04 (19.83)	27.26 (21.80)					
Feeling clear of values subscale					0.03 (0.20)	0.861	−0.10	0.00	−0.01
Intervention group^f^	45.11 (19.16)	27.51 (17.95)	31.79 (18.80)	30.69 (19.51)					
Control group	45.77 (19.67)	30.21 (16.63)	32.29 (21.08)	31.23 (21.26)					
Feeling supported subscale					0.15 (0.18)	0.384	−0.21	−0.11	−0.29
Intervention group^f^	41.14 (14.93)	22.20 (16.16)	23.61 (17.03)	22.12 (17.56)					
Control group	42.07 (14.01)	26.19 (19.31)	26.41 (22.59)	27.10 (20.03)					

a Raw means and SD are reported.

b Scores on all scales range from 0 to 100, with higher scores reflecting more decisional conflict.

c One missing value in the intervention group, *n* = 113, *n* = 108, and *n* = 104 for T1, T2, and T3, respectively.

d Effect size was calculated by the estimated marginal means and pooled SD (eg, mean_intervention group T1_-mean_intervention group T0_) - (mean_control group T1_-mean_control group T0_)/pooled SD_T0_).

e Calculated by summing 12 items (without 4 items of the effective decision-making subscale), dividing by 12, and multiplying with 25.

f Intervention group is reference group.

g Final model also included potential nonignorable dropout.

**Table 4.** Between-Group Effect in Decisional Conflict (Primary Outcome) over Time^a,b^

	T0^g^	T1^c,d^	T2^c^	T3^c^	T1			T2			T3		
	M (SD)	M (SD)	M (SD)	M (SD)	β (SE)	*P*	ES^e^	β (SE)	*P*	ES^e^	β (SE)	*P*	ES^e^
Total score					1.55 (1.91)	0.417	−0.11	0.41 (2.10)	0.847	−0.03	2.10 (2.23)	0.348	−0.13
Intervention group^f^		22.56 (13.96)	26.71 (14.20)	26.04 (15.38)									
Control group		24.17 (14.00)	27.50 (17.10)	28.08 (17.61)									
Effective decision-making subscale					−0.27 (2.40)	0.911	0.02	1.59 (2.40)	0.506	−0.09	2.63 (2.79)	0.347	−0.13
Intervention group^f^		17.79 (17.15)	22.11 (17.03)	22.66 (19.94)									
Control group		17.60 (17.88)	24.11 (18.70)	25.53 (21.22)									

a Raw means and SD are reported.

b Scores on all scales range from 0–100, with higher scores reflecting more decisional conflict.

c One missing value in the intervention group, *n* = 113, *n* = 108, and *n* = 104 for T1, T2, and T3, respectively.

d Sixteen missing values (7 intervention group, 9 control group) on total score and effective decision-making subscale as patients chose “not applicable” for >1 item of effective decision-making subscale, such that *n* = 106 in the intervention group and *n* = 103 in the control group.

e Effect size was calculated by the estimated marginal means and pooled SD (eg, mean_intervention group Tx_- mean_control group Tx_/pooled SD_Tx_).

f Intervention group is reference group.

g Items of the effective decision-making subscale were not assessed at baseline as these were considered inappropriate to assess before patients had a consultation with a plastic surgeon. Therefore, a total score (based on all 16 items) was not calculated.

### Secondary Outcomes

Results on continuous secondary outcomes are shown in Table 5 (descriptives) and Table 6 (group effects), and categorical secondary outcomes are presented in Table 7 (descriptives) and Table 8 (group effects).

**Table 5.** Descriptives of Secondary Outcomes over Time

		T0		T1		T2		T3
	No.	Mean (SD)	No.	Mean (SD)	No.	Mean (SD)	No.	Mean (SD)
Decision-making process								
Satisfaction with information (BREAST-Q)^a^								
Intervention group					80	65.75 (13.84)	85	64.84 (14.12)
Control group					80	63.11 (15.91)	81	63.48 (17.41)
Satisfaction with plastic surgeon (BREAST-Q)								
Intervention group			114	83.39 (18.13)				
Control group^b^			108	83.44 (17.86)				
Preparedness for decision-making^c^								
Intervention group			107	63.11 (26.45)				
Control group			106	52.51 (23.67)				
Perceived shared decision-making (SDM-Q-9)								
Intervention group			114	67.39 (20.97)				
Control group^b^			108	63.74 (19.07)				
Decision quality								
Knowledge								
Intervention group	126	7.06 (2.19)	114	8.92 (1.40)	109	8.80 (1.59)	105	8.54 (1.80)
Control group	124	6.88 (2.01)	112	8.60 (1.59)	111	8.68 (1.45)	107	8.08 (1.80)
Decision regret (DRS)								
Intervention group^d^					108	17.45 (17.19)	105	20.19 (17.32)
Control group					112	19.02 (18.60)	107	23.22 (19.89)
Patient-reported health outcomes								
Satisfaction with breasts (BREAST-Q)								
Intervention group^d,e^					108	51.72 (18.32)	104	55.70 (18.28)
Control group					112	52.83 (17.95)	107	57.23 (18.46)
Satisfaction with reconstruction outcomes (BREAST-Q)^a^								
Intervention group					80	62.88 (19.18)	86	64.84 (14.12)
Control group					81	57.93 (18.67)	82	63.48 (24.04)
Body image (QLQ-BR23)								
Intervention group					109	66.51 (27.68)	105	68.81 (28.12)
Control group					111	66.22 (28.97)	107	70.48 (28.67)
Sexual functioning (QLQ-BR23)								
Intervention group					109	25.69 (24.48)	105	26.35 (23.66)
Control group					111	26.58 (23.82)	107	29.75 (23.24)
Sexual enjoyment (QLQ-BR23)^f^								
Intervention group^g^					57	58.48 (26.93)	61	66.12 (23.95)
Control group^h^					64	58.85 (27.69)	70	62.38 (27.17)
Breast symptoms (QLQ-BR23)								
Intervention group					109	23.32 (17.85)	105	17.94 (18.84)
Control group					111	26.65 (20.62)	107	21.42 (21.14)
Anxiety (STAI-6)^i^								
Intervention group	126	47.88 (12.90)	114	45.58 (13.31)	109	40.86 (11.24)	105	39.30 (11.47)
Control group	124	44.87 (12.79)	112	43.87 (13.10)	111	38.89 (11.36)	107	37.51 (12.46)

SDM-Q-9, Shared Decision-Making Questionnaire 9 Items; DRS, Decision Regret Scale; QLQ-BR23, European Organisation of Research and Treatment of Cancer Breast Cancer Specific Quality of Life questionnaire; STAI-6, 6-Item Short-Form of the State Scale of the Spielberger State-Trait Anxiety Inventory.

a Only assessed in participants who had breast reconstruction.

b Four missing values (patients cancelled their consultation with a plastic surgeon).

c Thirteen missing (7 intervention group, 6 control group) (reasons: participant did not use pDA/information leaflet [*n* = 5], administrative mistake [*n* = 1], >2 items were answered with “not applicable” [*n* = 7]).

d 1 missing at T2.

e 1 missing at T3.

f Only assessed in participants who reported to have had some level of sexual activity in past 4 weeks (T2, *n* = 128; T3, *n* = 135).

g Three and 2 patients chose “not applicable” at T2 and T3, respectively, and were considered missing.

h Four and 2 patients chose “not applicable” at T2 and T3, respectively, and were considered missing.

i Final model also included random slope.

**Table 6.** Group Effects in Decision-Making Process, Decision Quality, and Patient-Reported Health Outcomes (Secondary Outcomes)^a^

	Between-Group Effect T1			Between-Group Effect T2			Between-Group Effect T3			Group by Time Effect		ES^b^		
	β (SE)	*P*	ES^c^	β (SE)	*P*	ES^c^	β (SE)	*P*	ES^c^	B (SE)	*P*	T0–T1	T0–T2	T0–T3
Decision-making process														
Satisfaction with information (BREAST-Q)^d,e^				−3.88 (2.27)	0.090	0.26	−2.87 (2.35)	0.223	0.18					
Satisfaction with plastic surgeon (BREAST-Q)^f^	0.01 (2.31)	0.996	0.00											
Preparedness for decision-making	−10.59 (3.42)	0.002^i^	0.42											
Perceived shared decision-making (SDM-Q-9)^f^	−3.88 (2.27)	0.090	0.18											
Decision quality														
Knowledge										0.00 (0.02)	0.954	0.05	−0.04	0.13
Decision regret (DRS)				1.52 (2.40)	0.527	−0.08	2.98 (2.52)	0.239	−0.16					
Patient-reported health outcomes														
Satisfaction with breasts (BREAST-Q)^f^				1.44 (2.31)	0.534	−0.08	1.36 (2.48)	0.585	−0.07					
Satisfaction with reconstruction outcomes (BREAST-Q)^d,e,g^				−6.87 (2.92)	0.020	0.36	−6.49 (3.29)	0.050	0.33					
Body image (QLQ-BR23)				−0.33 (3.79)	0.930	0.01	1.51 (3.84)	0.694	−0.05					
Sexual functioning (QLQ-BR23)				1.19 (3.24)	0.714	−0.05	3.11 (3.17)	0.328	−0.13					
Sexual enjoyment (QLQ-BR23)^h^				0.30 (4.84)	0.950	−0.01	−3.09 (4.43)	0.486	0.12					
Breast symptoms (QLQ-BR23)				3.31 (2.59)	0.202	−0.17	3.12 (2.73)	0.254	−0.16					
Anxiety (STAI-6)										0.11 (0.09)	0.204	−0.12	−0.11	−0.13

SDM-Q-9, Shared Decision-Making Questionnaire 9 Items; DRS, Decision Regret Scale; QLQ-BR23, European Organisation of Research and Treatment of Cancer Breast Cancer Specific Quality of Life questionnaire; STAI-6, 6-Item Short-Form of the State Scale of the Spielberger State-Trait Anxiety Inventory.

a The intervention group is the reference group.

b Effect size was calculated by the estimated marginal means and pooled SD (eg, mean_intervention group T1_-mean_intervention group T0_) - (mean_control group T1_-mean_control group T0_)/pooled SD_T0_).

c Effect size was calculated by the estimated marginal means and pooled SD (eg, mean_intervention group Tx_- mean_control group Tx_/pooled SD_Tx_).

d Only assessed in participants who had breast reconstruction.

e Final model also included baseline anxiety.

f Final model also included hospital.

g Final model also included BMI.

h Only assessed in participants who reported to have had some level of sexual activity in past 4 weeks (T2, *n* = 128; T3, *n* = 135).

i Significant effects.

**Table 7.** Descriptives of Categorical Secondary Outcomes over Time

	T1		T2		T3	
	Intervention Group (%)	Control Group (%)	Intervention Group (%)	Control Group (%)	Intervention Group (%)	Control Group (%)
Decision-making process						
Satisfaction with information in pDA or information leaflet	5 (4.4)	14 (12.5)				
Neutral	19 (16.7)	16 (14.3)				
Satisfied/very satisfied	86 (75.4)	80 (71.4)				
Missing	4 (3.5)	2 (1.8)				
Satisfaction with information about breast reconstruction						
Not at all satisfied/ not satisfied			3 (2.8)	6 (5.4)	3 (2.9)	10 (9.4)
Neutral			11 (10.1)	24 (21.4)	16 (15.2)	17 (15.9)
Satisfied/very satisfied			95 (87.2)	82 (73.2)	86 (81.9)	80 (74.8)
Perceived levels of involvement in decision-making						
Active	78 (68.4)	67 (59.8)				
Collaborative	15 (13.2)	24 (21.4)				
Passive	6 (5.3)	9 (8.0)				
Missing	15 (13.2)	12 (10.7)				
Patient-reported health outcomes						
Actual choice						
Immediate breast reconstruction^a^						
No			33 (29.7)	31 (27.7)		
Yes			78 (70.3)	81 (72.3)		
Type of immediate breast reconstruction^a^						
Tissue-expander			16 (20.5)	19 (23.5)		
Implant			57 (73.1)	51 (63.0)		
Autologous			3 (3.8)	8 (9.9)		
Combination implant and autologous			2 (2.6)	3 (3.7)		

a Patient-reported on T2. For 2 patients with missing data on T2, patient-reported data on T3 were used, such that *n* = 223.

**Table 8.** Group Differences in Secondary Categorical Outcomes^a^

	T1			T2			T3		
	β (SE)	χ^2^	*P*	β (SE)	χ^2^	*P*	β (SE)	χ^2^	*P*
Decision-making process									
Satisfaction with information in pDA or information leaflet	−0.37 (0.31)	1.42	0.233						
Satisfaction with information about breast reconstruction				−0.90 (0.36)	6.40	0.011	−0.48 (0.34)	2.01	0.157
Perceived levels of involvement in decision-making	−0.59 (0.32)	3.34	0.068						
Patient-reported health outcomes									
Actual choice									
Immediate breast reconstruction (no/yes)^b^				−0.10 (0.30)	0.12	0.735			
Type of immediate breast reconstruction (alloplastic/autologous)^b,c^				1.01 (0.70)	2.09	0.148			

SE standard error.

a Wald χ^2^ are reported for all outcomes.

b Patient-reported on T2 (for 2 patients with missing data on T2, patient-reported data on T3 were used).

c Alloplastic reconstruction includes reconstruction with tissue-expander, implant, and a combination of an implant and autologous tissue.

#### Decision-Making Process

Intervention group participants reported feeling better prepared for decision-making than those in the control group (preparedness for decision-making: ES_T1_ = 0.42, *P* = 0.002) (Table 6). There were no significant differences between the intervention and control groups in terms of their satisfaction with the plastic surgeon, perceived levels of shared decision-making during consultation with their plastic surgeon, satisfaction with information about BR, satisfaction with information in the pDA or the information leaflet at T1, and the perceived levels of involvement in decision-making. In women who received BR, satisfaction with information (measured with the BREAST-Q) did not differ between the intervention and control groups, and remained stable over time. (**See Table, Supplemental Digital Content 3**, which shows the effects of time on secondary outcomes, http://links.lww.com/PRS/G978.)

#### Decision Quality

In both groups, knowledge of BR significantly increased from baseline to T1 (linear time effect: β [SE] = 0.07 [0.01], *P* < 0.001), and remained stable during T2 and T3 (Tables 5 and 6 and **Supplemental Digital Content 3**, http://links.lww.com/PRS/G978). There were no between-group differences in knowledge of BR over time or in decision regret at T2 and T3 (Tables 5 and 6). At T3, 34.0% of all participants experienced clinically relevant levels of decision regret (score ≥30) (no between-group difference: chi-square, 1.16, *P* = 0.561).

#### Patient-Reported Health Outcomes

At T2 and T3, no differences were found between the intervention and control groups in terms of satisfaction with breasts, satisfaction with reconstruction outcome (in women who received BR), body image, sexual functioning, sexual enjoyment, and breast symptoms. There were no significant differences between groups in anxiety over time; in both groups, anxiety decreased significantly over time (linear time effect: β [SE] = −0.45 [0.06], *P* = 0.000). In both groups, breast symptoms significantly decreased from T2 to T3 (*P* = 0.005). There were no significant time effects from T2 to T3 in any other patient-reported health outcome. The actual choice of whether or not to have IBR and regarding the type of BR did not differ between groups (Tables 7 and 8). The majority had IBR (70.3% and 72.3% for the intervention group and the control group, respectively).

## DISCUSSION

This study aimed to evaluate the efficacy of an online pDA in reducing decisional conflict in women considering IBR. Both the pDA and the information leaflet were effective in reducing decisional conflict. The pDA, however, provided additional improvement over CAU in the decision-making process by enabling patients to feel better prepared for making a decision. No added value of the pDA over CAU was found on other outcomes related to the decision-making process, decision quality and health outcomes.

The benefit of the pDA in improving patients’ preparedness for decision-making is in line with health care professionals’ expectations that a BR pDA would help patients to prepare for consultation,^45^ and the qualitative experiences of both patients and health care professionals with using a BR pDA.^65,66^ Our finding that the pDA did not affect patients’ anxiety is in line with existing literature,^40,42^ and is important given the concern that shared decision-making can unintentionally increase anxiety in patients.^67,68^

The lack of any beneficial effect of our pDA over CAU on other outcomes related to the decision-making process and decision quality seems in stark contrast with the body of evidence showing the beneficial effects of pDAs in all kinds of health care decisions, including decisions about BR.^40,42,43,69,70^ It might be that in our study the effects of the pDA are underestimated, as the CAU control group received an information leaflet. Although this information leaflet is widely available in Dutch hospitals and on the internet, the active provision of the leaflet to the control group before their consultation with a plastic surgeon might have led to higher uptake and possibly more profound processing of the information in the leaflet. This could have positively benefitted the decision-making process in that the information led to decreased decisional conflict, increased knowledge about BR, and higher perceived levels of involvement in decision-making, more than in a true CAU setting. However, given the substantial time and effort that was required of all participants in this trial, including the control group, we provided the information leaflet to the control group for ethical reasons. In addition, most women in both groups used the internet (almost) daily. This may also have had an impact on decision-making, and may partly explain the minimal differences between the two groups. Also, study participation itself might have increased awareness for the importance of information provision and shared decision-making about IBR among patients and health care professionals, leading to contamination bias.

This study had some limitations. First, our sample was relatively young and highly educated, limiting the generalizability of our findings. Second, although we assume that randomization successfully led to two comparable groups, the lack of baseline assessment of some outcomes (ie, satisfaction with information, body image, sexual functioning, breast symptoms) limits our conclusions. Although some outcomes were not considered appropriate at baseline (such as decision regret, and preparation for decision-making), others were omitted to limit burden for participants. Furthermore, our study lacks observations of the interaction that took place between patients and their physicians during consultation (eg, by audio recordings of consultations). Adding such observations could provide more detailed insights into the effect of the pDA on the shared decision-making process.^71^ Strengths of this study include the randomized controlled trial design of our study, the long follow-up, the high participation rate, and the low attrition rate.

For future studies, an even longer-term follow-up assessment (>12 months) could provide more insights into the effect of the pDA on outcomes such as decision regret, satisfaction with breasts, and satisfaction with reconstruction outcome, given the lengthy recovery process of BR and additional procedures that are often required after BR. Also, an extra assessment before consultation with a plastic surgeon (and after pDA use) would allow to better distinguish effects of the pDA from the effects of the consultation itself. This time point seems especially interesting, as our results show that patients felt better prepared for consultation by the pDA.

## CONCLUSIONS

Our findings indicate that both the online pDA and the information leaflet are helpful for breast cancer patients having to make a decision about IBR. The online pDA better prepares patients for consultation with their plastic surgeon and decision-making than an information leaflet. Also, the online format of the pDA more easily allows for adaptions required by future developments in BR options and scientific evidence, and for the further tailoring of information to patients’ personal situation and information needs. Potential benefits in cost-effectiveness of the pDA, including decreased health care use, and the preferences among health care providers should be further investigated. Altogether, we recommend the pDA for use in clinical practice.

## DISCLOSURE

After trial completion, ZorgKeuzeLab will request user fees from hospitals to implement the decision aid. Under certain conditions, the NKI-AVL will receive a percentage of these user fees. The authors have no financial interest in any of the products mentioned in this article, and have no relevant conflicts of interest to report.

## ACKNOWLEDGMENTS

This study was funded by the Dutch Cancer Society (grant no. A6C/NKI 2014–7031). Research at the Netherlands Cancer Institute is supported by institutional grants of the Dutch Cancer Society and of the Dutch Ministry of Health, Welfare, and Sport. The authors would like to thank all patients for participating in this study, all health care professionals that contributed to the administration of the study or the recruitment of participants, and Regina The and Klemens Karssen (ZorgKeuzeLab) for collaborating on the development and implementation of the decision aid. Furthermore, they are grateful to Dr. Ellen Engelhardt for suggestions on how to improve their article.

## Supplementary Material



## References

[1] Ferlay J Ervik M Lam F. Global cancer observatory: cancer today. Lyon, France: International Agency for Research on Cancer; 2020. Available at: https://gco.iarc.fr/today. Accessed July 15, 2022.

[2] Jeevan R Cromwell DA Browne JP. Findings of a national comparative audit of mastectomy and breast reconstruction surgery in England. J Plast Reconstr Aesthet Surg. 2014;67:1333–1344. 24908545 10.1016/j.bjps.2014.04.022

[3] Roder D Zorbas H Kollias J. Factors predictive of immediate breast reconstruction following mastectomy for invasive breast cancer in Australia. Breast 2013;22:1220–1225. 24128741 10.1016/j.breast.2013.09.011

[4] National Breast and Ovarian Cancer Centre. National Breast and Ovarian Cancer Centre and Royal Australasian College of Surgeons National Breast Cancer Audit Public Health Monitoring Series 2008 Data. Surry Hills, Australia: National Breast and Ovarian Cancer Centre; 2010.

[5] NABON Breast Cancer Audit. NBCA annual report 2017. Available at: https://dica.nl/jaarrapportage-2017. Accessed January 7, 2019.

[6] Parker PA Youssef A Walker S. Short-term and long-term psychosocial adjustment and quality of life in women undergoing different surgical procedures for breast cancer. Ann Surg Oncol. 2007;14:3078–3089. 17574501 10.1245/s10434-007-9413-9

[7] Koçan S Gürsoy A. Body image of women with breast cancer after mastectomy: a qualitative research. J Breast Health 2016;12:145–150. 28331752 10.5152/tjbh.2016.2913 PMC5351438

[8] Janni W Rjosk D Dimpfl T. Quality of life influenced by primary surgical treatment for stage I-III breast cancer—long-term follow-up of a matched-pair analysis. Ann Surg Oncol. 2001;8:542–548. 11456055 10.1007/s10434-001-0542-2

[9] Chen CL Liao MN Chen SC Chan PL Chen SC. Body image and its predictors in breast cancer patients receiving surgery. Cancer Nurs. 2012;35:E10–E16. 22067694 10.1097/NCC.0b013e3182336f8b

[10] Lee GK Sheckter CC. Breast reconstruction following breast cancer treatment-2018. JAMA 2018;320:1277–1278. 30178060 10.1001/jama.2018.12190

[11] Dutch Society for Plastic Surgery. Guideline breast reconstruction. Available at: https://richtlijnendatabase.nl/en/richtlijn/breast_reconstruction/breast_reconstruction_after_mastectomy.html. Accessed January 1, 2023.

[12] National Breast Cancer Network Netherlands. Breast cancer guideline. Available at: https://www.nabon.nl/wp-content/uploads/2022/10/Dutch-Breast-Cancer-Guideline-2012.pdf. Accessed January 1, 2023.

[13] Panchal H Matros E. Current trends in postmastectomy breast reconstruction. Plast Reconstr Surg. 2017;140(Advances in Breast Reconstruction):7S–13S. 10.1097/PRS.0000000000003941 PMC5722225 29064917

[14] Mennie JC Mohanna PN O’Donoghue JM Rainsbury R Cromwell DA. National trends in immediate and delayed post-mastectomy reconstruction procedures in England: a seven-year population-based cohort study. Eur J Surg Oncol. 2017;43:52–61. 27776942 10.1016/j.ejso.2016.09.019

[15] van Bommel A Spronk P Mureau M. Breast-contour-preserving procedure as a multidisciplinary parameter of esthetic outcome in breast cancer treatment in the Netherlands. Ann Surg Oncol. 2019;26:1704–1711. 30830541 10.1245/s10434-019-07265-3 PMC6510878

[16] Kamali P van Bommel A Becherer B. Immediate breast reconstruction in the Netherlands and the United States: a proof-of-concept to internationally compare quality of care using cancer registry data. Plast Reconstr Surg. 2019;144:565e–574e. 10.1097/PRS.0000000000006011 31568284

[17] Lang JE Summers DE Cui H. Trends in post-mastectomy reconstruction: a SEER database analysis. J Surg Oncol. 2013;108:163–168. 23861196 10.1002/jso.23365 PMC4035023

[18] Jeevan R Mennie JC Mohanna PN O’Donoghue JM Rainsbury RM Cromwell DA. National trends and regional variation in immediate breast reconstruction rates. Br J Surg. 2016;103:1147–1156. 27324317 10.1002/bjs.10161

[19] NABON Breast Cancer Audit. DICA annual report 2021. Available at: https://dica.nl/. Accessed January 15, 2023.

[20] van Egdom LSE de Ligt KM de Munck L. Predictors of delayed breast reconstruction in the Netherlands: a 5-year follow-up study in stage I-III breast cancer patients. Breast Cancer 2022;29:324–335. 34780034 10.1007/s12282-021-01313-1 PMC8885490

[21] Hvilsom GB Hölmich LR Frederiksen K Steding-Jessen M Friis S Dalton SO. Socioeconomic position and breast reconstruction in Danish women. Acta Oncol. 2011;50:265–273. 21091086 10.3109/0284186X.2010.529823

[22] National Clinical Audit Support Programme (NCASP). National mastectomy and breast reconstruction audit 2009. The Information Centre (NHS); 2009. Available at: https://files.digital.nhs.uk/publicationimport/pub02xxx/pub02722/clin-audi-supp-prog-mast-brea-reco-2009-rep1.pdf. Accessed January 1, 2024.

[23] van Bommel AC Mureau MAM Schreuder K. Large variation between hospitals in immediate breast reconstruction rates after mastectomy for breast cancer in the Netherlands. J Plast Reconstr Aesthet Surg. 2017;70:215–221. 27993547 10.1016/j.bjps.2016.10.022

[24] Jagsi R Jiang J Momoh AO. Trends and variation in use of breast reconstruction in patients with breast cancer undergoing mastectomy in the United States. J Clin Oncol. 2014;32:919–926. 24550418 10.1200/JCO.2013.52.2284 PMC4876312

[25] Dutch Institute for Cancer Auditing. NABON Breast Cancer Audit (NBCA) Annual Report Results 2019. Published October 16, 2020.

[26] Sheehan J Sherman KA Lam T Boyages J. Association of information satisfaction, psychological distress and monitoring coping style with post-decision regret following breast reconstruction. Psychooncology 2007;16:342–351. 16874745 10.1002/pon.1067

[27] Dikmans REG van de Grift TC Bouman MB Pusic AL Mullender MG. Sexuality, a topic that surgeons should discuss with women before risk-reducing mastectomy and breast reconstruction. Breast 2019;43:120–122. 30550924 10.1016/j.breast.2018.12.003

[28] Kuo NT Kuo YL Lai HW Ko NY Fang SY. The influence of partner involvement in the decision-making process on body image and decision regret among women receiving breast reconstruction. Support Care Cancer 2019;27:1721–1728. 30132239 10.1007/s00520-018-4416-6

[29] Lee CN Pignone MP Deal AM. Accuracy of predictions of patients with breast cancer of future well-being after immediate breast reconstruction. JAMA Surg. 2018;153:e176112. 29417143 10.1001/jamasurg.2017.6112 PMC5875341

[30] Hasak JM Myckatyn TM Grabinski VF Philpott SE Parikh RP Politi MC. Stakeholders’ perspectives on postmastectomy breast reconstruction: recognizing ways to improve shared decision making. Plast Reconstr Surg Glob Open 2017;5:e1569. 29263969 10.1097/GOX.0000000000001569 PMC5732675

[31] Zhong T Hu J Bagher S. Decision regret following breast reconstruction: the role of self-efficacy and satisfaction with information in the preoperative period. Plast Reconstr Surg. 2013;132:724e–734e. 10.1097/PRS.0b013e3182a3bf5d 24165624

[32] Potter S Mills N Cawthorn S Wilson S Blazeby J. Exploring information provision in reconstructive breast surgery: a qualitative study. Breast 2015;24:732–738. 26422125 10.1016/j.breast.2015.09.003

[33] Soon PS Ruban S Mo HTJ. Understanding patient choices regarding breast reconstruction after mastectomy for breast cancer. Support Care Cancer 2019;27:2135–2142. 30251065 10.1007/s00520-018-4470-0

[34] Flitcroft K Brennan M Spillane A. Decisional regret and choice of breast reconstruction following mastectomy for breast cancer: a systematic review. Psychooncology 2018;27:1110–1120. 29143481 10.1002/pon.4585

[35] Zhong T Bagher S Jindal K. The influence of dispositional optimism on decision regret to undergo major breast reconstructive surgery. J Surg Oncol. 2013;108:526–530. 24105811 10.1002/jso.23437

[36] Manne SL Topham N Kirstein L. Attitudes and decisional conflict regarding breast reconstruction among breast cancer patients. Cancer Nurs. 2016;39:427–436. 26780376 10.1097/NCC.0000000000000320 PMC4947023

[37] Fallbjörk U Frejeus E Rasmussen BH. A preliminary study into women’s experiences of undergoing reconstructive surgery after breast cancer. Eur J Oncol Nurs. 2012;16:220–226. 21764374 10.1016/j.ejon.2011.05.005

[38] Murray CD Turner A Rehan C Kovacs T. Satisfaction following immediate breast reconstruction: experiences in the early post-operative stage. Br J Health Psychol. 2015;20:579–593. 24946693 10.1111/bjhp.12112

[39] Joseph-Williams N Newcombe R Politi M. Toward minimum standards for certifying patient decision aids: a modified Delphi consensus process. Med Decis Making 2014;34:699–710. 23963501 10.1177/0272989X13501721

[40] Stacey D Légaré F Lewis K. Decision aids for people facing health treatment or screening decisions. Cochrane Database Syst Rev. 2017;4:CD001431. 28402085 10.1002/14651858.CD001431.pub5 PMC6478132

[41] Sheehan J Sherman KA. Computerised decision aids: a systematic review of their effectiveness in facilitating high-quality decision-making in various health-related contexts. Patient Educ Couns. 2012;88:69–86. 22185961 10.1016/j.pec.2011.11.006

[42] Paraskeva N Guest E Lewis-Smith H Harcourt D. Assessing the effectiveness of interventions to support patient decision making about breast reconstruction: a systematic review. Breast 2018;40:97–105. 29730304 10.1016/j.breast.2018.04.020

[43] Berlin NL Tandon VJ Hawley ST. Feasibility and efficacy of decision aids to improve decision making for postmastectomy breast reconstruction: a systematic review and meta-analysis. Med Decis Making 2019;39:5–20. 30799692 10.1177/0272989X18803879

[44] ter Stege JA Woerdeman LAE Hahn DEE. The impact of an online patient decision aid for women with breast cancer considering immediate breast reconstruction: study protocol of a multicenter randomized controlled trial. BMC Med Inform Decis Mak. 2019;19:165. 31426772 10.1186/s12911-019-0873-1 PMC6701008

[45] Ter Stege JA Raphael DB Oldenburg HSA. Development of a patient decision aid for patients with breast cancer who consider immediate breast reconstruction after mastectomy. Health Expect. 2022;25:232–244. 34708487 10.1111/hex.13368 PMC8849254

[46] Dutch Cancer Society. Breast reconstruction [in Dutch]. Available at: https://www.kanker.nl/sites/default/files/library_files/526/brochure-Borstreconstructie.pdf. Accessed July 1, 2017.

[47] Degner LF Sloan JA Venkatesh P. The control preferences scale. Can J Nurs Res. 1997;29:21–43. 9505581

[48] van Zuuren FJ de Groot KI Mulder NL Muris P. Coping with medical threat: an evaluation of the threatening medical situations inventory (TMSI). Pers Individ Dif. 1996;21:21–31.

[49] O’Connor AM. Validation of a decisional conflict scale. Med Decis Making 1995;15:25–30. 7898294 10.1177/0272989X9501500105

[50] Koedoot N Molenaar S Oosterveld P. The decisional conflict scale: further validation in two samples of Dutch oncology patients. Patient Educ Couns. 2001;45:187–193. 11722854 10.1016/s0738-3991(01)00120-3

[51] O’Connor AM. User Manual—Decisional Conflict Scale. Ottawa, Ontario, Canada: Ottawa Hospital Research Institute; 1993.

[52] Pusic AL Klassen AF Scott AM Klok JA Cordeiro PG Cano SJ. Development of a new patient-reported outcome measure for breast surgery: the BREAST-Q. Plast Reconstr Surg. 2009;124:345–353. 19644246 10.1097/PRS.0b013e3181aee807

[53] Graham ID O’Connor AM. User Manual—Preparation for Decision Making Scale (document on the internet). Ottawa, Ontario, Canada: Ottawa Hospital Research Institute; 1995.

[54] Bennett C Graham ID Kristjansson E Kearing SA Clay KF O’Connor AM. Validation of a preparation for decision making scale. Patient Educ Couns. 2010;78:130–133. 19560303 10.1016/j.pec.2009.05.012

[55] Kriston L Scholl I Holzel L Simon D Loh A Harter M. The 9-item Shared Decision Making Questionnaire (SDM-Q-9). Development and psychometric properties in a primary care sample. Patient Educ Couns. 2010;80:94–99. 19879711 10.1016/j.pec.2009.09.034

[56] Rodenburg-Vandenbussche S Pieterse AH Kroonenberg PM. Dutch translation and psychometric testing of the 9-item Shared Decision Making Questionnaire (SDM-Q-9) and Shared Decision Making Questionnaire-physician version (SDM-Q-doc) in primary and secondary care. PLoS One 2015;10:e0132158. 26151946 10.1371/journal.pone.0132158 PMC4494856

[57] Brehaut JC O’Connor AM Wood TJ. Validation of a decision regret scale. Med Decis Making 2003;23:281–292. 12926578 10.1177/0272989X03256005

[58] O’Connor AM. User Manual—Decision Regret Scale (document on the internet). Ottawa, Ontario, Canada: Ottawa Hospital Research Institute; 1996.

[59] Sprangers MA Groenvold M Arraras JI. The European Organization for Research and Treatment of Cancer breast cancer-specific quality-of-life questionnaire module: first results from a three-country field study. J Clin Oncol. 1996;14:2756–2768. 8874337 10.1200/JCO.1996.14.10.2756

[60] Marteau TM Bekker H. The development of a six-item short-form of the state scale of the Spielberger State-Trait Anxiety Inventory (STAI). Br J Clin Psychol. 1992;31:301–306. 1393159 10.1111/j.2044-8260.1992.tb00997.x

[61] Schwarz G. Estimating the dimension of a model. Ann Stat. 1978;6:461–464.

[62] Akaike H. Information theory and an extension of the maximum likelihood principle. In: Parzen E. Tanabe K Kitagawa G, eds. Selected Papers of Hirotugu Akaike. New York: Springer; 1998:199–213.

[63] Cohen J. Statistical Power Analysis for the Behavioral Sciences. Vol. 2, 2nd ed. Hillsdale, NJ: Lawrence Earlbaum Associates; 1988.

[64] Norman GR Sloan JA Wyrwich KW. Interpretation of changes in health-related quality of life: the remarkable universality of half a standard deviation. Med Care 2003;41:582–592. 12719681 10.1097/01.MLR.0000062554.74615.4C

[65] Sherman KA Shaw LK Jørgensen L. Qualitatively understanding patients’ and health professionals’ experiences of the BRECONDA breast reconstruction decision aid. Psychooncology 2017;26:1618–1624. 27957772 10.1002/pon.4346

[66] Sherman KA Harcourt DM Lam TC Shaw LK Boyages J. BRECONDA: development and acceptability of an interactive decisional support tool for women considering breast reconstruction. Psychooncology 2014;23:835–838. 24991748 10.1002/pon.3498

[67] Coulter A. Partnerships with patients: the pros and cons of shared clinical decision-making. J Health Serv Res Policy 1997;2:112–121. 10180362 10.1177/135581969700200209

[68] Caldon LJ Collins KA Reed MW.; BresDex Group. Clinicians’ concerns about decision support interventions for patients facing breast cancer surgery options: understanding the challenge of implementing shared decision-making. Health Expect. 2011;14:133–146. 21029281 10.1111/j.1369-7625.2010.00633.x PMC5060572

[69] Yang S Yu L Zhang C. Effects of decision aids on breast reconstruction: a systematic review and meta-analysis of randomised controlled trials. J Clin Nurs. 2022;32:1025–1044. 35460127 10.1111/jocn.16328

[70] Mardinger C Steve AK Webb C Sherman KA Temple-Oberle C. Breast reconstruction decision aids decrease decisional conflict and improve decisional satisfaction: a randomized control trial. Plast Reconstr Surg. 2022;151:278–288. 36696307 10.1097/PRS.0000000000009830

[71] Scholl I Kriston L Dirmaier J Härter M. Comparing the nine-item Shared Decision-Making Questionnaire to the OPTION scale—an attempt to establish convergent validity. Health Expect. 2015;18:137–150. 23176071 10.1111/hex.12022 PMC5060753

[72] Reumkens K Tummers MHE Severijns Y. Reproductive decision-making in the context of hereditary cancer: the effects of an online decision aid on informed decision-making. J Community Genet. 2021;12:101–110. 32880035 10.1007/s12687-020-00484-2 PMC7846643

[73] Garvelink MM Boland L Klein K. Decisional conflict scale use over 20 years: the anniversary review. Med Decis Making 2019;39:301–314. 31142194 10.1177/0272989X19851345

[74] Sherman KA Kilby CJ Shaw LK. Facilitating decision-making in women undergoing genetic testing for hereditary breast cancer: BRECONDA randomized controlled trial results. Breast 2017;36:79–85. 29031121 10.1016/j.breast.2017.10.001

[75] Becerra Perez MM Menear M Brehaut JC Légaré F. Extent and predictors of decision regret about health care decisions: a systematic review. Med Decis Making 2016;36:777–790. 26975351 10.1177/0272989X16636113

