# Supplementary material for: Efficacy of a Decision Aid in Breast Cancer Patients Considering Immediate Reconstruction: Results of a Randomized Controlled Trial
Source: Plast Reconstr Surg. 2023 Oct 9;154(4):706–22. doi: 10.1097/PRS.0000000000011100 (PMC11412569; doi:10.1097/PRS.0000000000011100)
Supplement: Supplementary file 1 [file prs-154-0706-s001.pdf]

Supplemental Digital Content 1. Table showing group differences in adjuvant treatment, complications of breast surgery, and nipple reconstruction.

	All Patients (N=212)[a]		Intervention Group (N=105)		Control Group (N=107)		
	N	%	N	%	N	%	*p*
Adjuvant treatment[b]							
Radiotherapy (yes)	71	33.5	33	31.4	38	35.5	.529
Chemotherapy (yes)	43	20.3	23	21.9	20	18.7	.561
Endocrine therapy (yes)	110	51.9	54	51.4	56	52.3	.895
Immunotherapy (yes)	22	10.4	14	13.3	8	7.5	.162
Complication(s) of breast surgery (yes)[b]	59	27.8	31	29.5	28	26.2	.586
Lost BR due to complication(s) (yes)[b*]	19	9.0	8	7.6	11	10.3	.498
Nipple reconstruction[b**]							
No, nipple was spared	65	38.7	30	34.9	35	42.7	.275
No, nipple was removed	92	54.8	52	60.5	40	48.8	
Yes	11	6.5	4	4.7	7	8.5	

Abbreviations: **BR** breast reconstruction.

[a]Selection of participants who completed T3.

[b]Patient-reported at 12 months after surgery (T3).

*12 patients who lost their BR due to complication(s) reported to have BR (again) at time of completing T3.

**Only assessed in participants who had BR (n=168, 86 in the intervention group, 82 in the control group).
