# Supplementary material for: Efficacy of a Decision Aid in Breast Cancer Patients Considering Immediate Reconstruction: Results of a Randomized Controlled Trial
Source: Plast Reconstr Surg. 2023 Oct 9;154(4):706–22. doi: 10.1097/PRS.0000000000011100 (PMC11412569; doi:10.1097/PRS.0000000000011100)
Supplement: Supplementary file 2 [file prs-154-0706-s002.pdf]

Supplemental Digital Content 2. Table showing the effects of time on decisional conflict.

	Linear Time effect			Quadratic Time effect		
	B	SE	*p*	B	SE	*p*
Decisional Conflict (DCS)						
Combined score without Effective Decision Making subscale[a]	-0.52	0.12	**.000**	0.01	0.00	**.002**
Uncertainty subscale	-0.37	0.15	.016	0.00	0.00	.090
Feeling Informed subscale	-0.77	0.17	**.000**	0.01	0.00	**.001**
Feeling Clear of Values subscale	-0.35	0.15	.015	0.00	0.00	.094
Feeling Supported subscale	-0.58	0.13	**.000**	0.01	0.00	**.002**
	T1-T2			T1-T3		
	B	SE	*p*	B	SE	*p*
Total score	4.36	1.49	**.004**	3.42	1.65	.040
Effective Decision Making subscale	4.44	2.01	.028	4.89	2.28	.033

Abbrevations: **B** beta; **SE** standard error; **DCS** decisional conflict scale.

T0 baseline; **T1** 1 week after consultation plastic surgeon; **T2** 3 months after surgery; **T3** 12 months after surgery.

[a]Calculated by summing 12 items (without 4 items of the Effective Decision Making subscale), dividing by 12, and multiplying with 25.

Intervention group is reference group.
