# Supplementary material for: Efficacy of a Decision Aid in Breast Cancer Patients Considering Immediate Reconstruction: Results of a Randomized Controlled Trial
Source: Plast Reconstr Surg. 2023 Oct 9;154(4):706–22. doi: 10.1097/PRS.0000000000011100 (PMC11412569; doi:10.1097/PRS.0000000000011100)
Supplement: Supplementary file 3 [file prs-154-0706-s003.pdf]

**Supplemental Digital Content 3.** Table showing the effects of time on secondary outcomes.

| | Linear Time effect | | | Quadratic Time effect | | | T2-T3 | | |
|---|---|---|---|---|---|---|---|---|---|
| | B | SE | *p* | B | SE | *p* | B | SE | *p* |
| *Decision-making process* | | | | | | | | | |
| Satisfaction with information (BREAST-Q)[a] | | | | | | | -0.45 | 1.53 | .770 |
| *Decision quality* | | | | | | | | | |
| Knowledge | 0.07 | 0.01 | **.000** | -0.00 | 0.00 | **.000** | | | |
| Decision regret (DRS) | | | | | | | 2.75 | 1.69 | .106 |
| *Patient-reported health outcomes* | | | | | | | | | |
| Satisfaction with breasts (BREAST-Q) | | | | | | | 4.35 | 1.83 | .018 |
| Satisfaction with outcomes (BREAST-Q)[a] | | | | | | | -0.79 | 2.20 | .720 |
| Body image (QLQ-BR23) | | | | | | | 1.79 | 1.95 | .361 |
| Sexual functioning (QLQ-BR23) | | | | | | | 1.04 | 2.08 | .619 |
| Sexual enjoyment (QLQ-BR23)[b] | | | | | | | 7.68 | 3.61 | .035 |
| Breast symptoms (QLQ-BR23) | | | | | | | -5.17 | 1.82 | **.005** |
| Anxiety (STAI-6) | -0.45 | 0.06 | **.000** | 0.01 | 0.00 | **.000** | | | |

Abbrevations: **B** beta; **SE** standard error; **DRS** decision regret scale; **QLQ-BR23** european organisation of research and treatment of cancer breast cancer specific quality of life questionaire; **STAI-6** six-item short-form of the state scale of the spielberger State-Trait Anxiety Inventory. **T2** 3 months after surgery; **T3** 12 months after surgery.

[a]Only assessed in participants who had breast reconstruction.

[b]Only assessed in participants who reported to have had some level of sexual activity in past 4 weeks (T2 n=128, T3 n=135).

Intervention group is the reference group.

Bold font indicates significant effects.
